# Investigating the Conformational Stability of Prion Strains through a Kinetic Replication Model

**DOI:** 10.1371/journal.pcbi.1000420

**Published:** 2009-07-03

**Authors:** Mattia Zampieri, Giuseppe Legname, Claudio Altafini

**Affiliations:** 1 Functional Analysis Sector, International School for Advanced Studies, Trieste, Italy; 2 Neurobiology Sector, International School for Advanced Studies, Trieste, Italy; Harvard University, United States of America

## Abstract

Prion proteins are known to misfold into a range of different aggregated forms, showing different phenotypic and pathological states. Understanding strain specificities is an important problem in the field of prion disease. Little is known about which PrP^Sc^ structural properties and molecular mechanisms determine prion replication, disease progression and strain phenotype. The aim of this work is to investigate, through a mathematical model, how the structural stability of different aggregated forms can influence the kinetics of prion replication. The model-based results suggest that prion strains with different conformational stability undergoing *in vivo* replication are characterizable *in primis* by means of different rates of breakage. A further role seems to be played by the aggregation rate (i.e. the rate at which a prion fibril grows). The kinetic variability introduced in the model by these two parameters allows us to reproduce the different characteristic features of the various strains (e.g., fibrils' mean length) and is coherent with all experimental observations concerning strain-specific behavior.

## Author Summary

Prion diseases are caused by the accumulation of a cellular prion protein with an altered conformation, which acts as a catalyst for the further recruitment and the modification of the normal form of the protein. Protein polymerization appears to have a central role in the progression of the disease, an aspect shared with several other neurodegenerative diseases. The aim of this work is to investigate at the kinetic level the “prion strain phenomenon”, i.e., the ability of prion proteins to misfold into a range of different aggregated forms exhibiting different replication and propagation properties. The dynamics of prion replication is investigated with the help of a mathematical model. We relate a measurement accessible *in vitro* (prion structural stability) to a mathematical description of the fibrils' kinetics *in vivo*. The analysis of the model suggests that the replication kinetics of the different prion strains is characterizable by means of two parameters, representing the rates of breakage and aggregation. This result is coherent with various experimental findings concerning strain-specific behavior, such as, for example, the observation of the fibril mean length of the various strains.

## Introduction

Prions are infectious agents composed solely of proteins, whose replication does not rely upon the presence of nucleic acids [1]. Although the molecular mechanisms of prion replication are poorly understood, the current working hypothesis is based on the assumption that prions replicate by means of an autocatalytic process which converts cellular prion protein (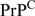) to the disease-associated misfolded PrP isoform (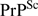). This process of replication of a prion depends upon the capacity of the pathogenic protein form to bind to and to catalyze the conversion of existing intermediate molecules. Recent studies [2] have observed that the prion protein can misfold into a range of different aggregated forms derived from a continuum of 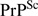 structural conformation templates [3] from which different phenotypic and pathological states derive. The ability of the same encoded protein to encipher a multitude of phenotypic states is known as the “prion strain phenomenon” [4]. Prion strains are defined as infectious isolates that, when transmitted to identical hosts, exhibit the following distinct prion disease phenotypes:

i) Proteinase K (PK) digestion profile;
ii) Incubation time;
iii) Histopathological lesion profiles;
iv) Specific neuronal target areas.

A reason for the strain phenomenon can be the association of 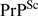 to several disease conformations, characterizable by means of a different stability against denaturation, different post-translational modifications (e.g. glycosylation) and distinct cleavage sites. These observations are reinforced by [5], where it is reported that the amyloid fibrils (formed by the 40-residues 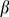-amyloid peptide) with different morphologies have significantly different molecular structures. These differences are shown to be self-propagating and to be associated with different toxicities, suggesting the possibility for a structural origin of prion strains. Moreover, recent studies on prion disease have confirmed that the incubation time is related not only to the inoculum dosage and the prion protein expression, but also to the resistance of prion strains against denaturation [3] in terms of the concentration of guanidine hydrochloride (Gdn-HCl) required to denaturate 50% of the disease-causing protein (see Text S1 for further discussions). Other studies have highlighted a strong relationship between the stability of the prion protein against denaturation and neuropathological lesion profiles [6],[7]. Lesions due to stable prions tend to show large vacuolations localized in specific small brain regions, whilst lesions due to unstable prion strains show a less intense vacuolation and are more widely distributed in the brain. Apart from these properties, crucial details of the molecular mechanisms enabling the characterization of different prion strains are still missing. For example, neither structural characterizations of 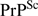, nor maps of protein-protein interactions have so far been provided, and even the 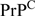 biological function is unclear. Hence, in order to use the existing data to gain some insight into the properties of the different prion strains, we decided to follow a model-based approach.

In this paper, using a well established model for the kinetics of the in vivo prion replication [8], we relate the evidence about conformational stability to the parameters of the model describing the evolution in time of the fibril length. The main points we deduce from our analysis are:

i) In terms of the model, the key parameter describing strain-dependent replication kinetics is the fibril breakage rate.
ii) A precise fitting of the model prediction to the experimental data is obtained assuming that also the aggregation rate changes with the strain. In particular, a functional dependence on the breakage rate is assumed.
iii) The prediction of the model is that the stability against denaturation is inversely correlated to both breakage and aggregation rates and directly correlated with the mean length of fibrils.
iv) By fitting experimental data, we can quantitatively predict the fibril length distributions associated to different prion strains.

Multiple experimental observations *in vitro* [9] and in yeast [10],[11] support our model-based considerations, reinforcing our predictions for *in vivo* mammalian systems.

## Results

Protein polymerization seems to have a central role in the progression of the prion pathology, an aspect shared with several other neurodegenerative diseases associated with different aggregating proteins, such as Alzheimer's (A 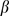), Parkinson's (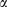-synuclein) and Huntington's (huntingtin) diseases. The aggregation kinetics of amyloid peptides has been studied extensively (see [12],[13]), and has shed light on the wide range of amyloid aggregation mechanisms observed. Many modeling approaches have been introduced for this purpose in recent years, e.g. theoretical models consisting of nonlinear ordinary differential equations (ODEs), two-dimensional lattice-based statistical models and molecular dynamics simulations [8], [13]–[18]. In this paper we explore a mathematical description of the prion replication dynamics through nonlinear ODEs. This class of models explain the appearance of the disease by means of a bistability induced by a quadratic term, as in classical epidemic models [19]. The model we used is drawn from [8],[14] and is based on a nucleated polymerization mechanism [20] (see Materials and Methods). This approach has been shown to overcome the limitations of the “heterodimer model” [1] and to be a reasonable simplification of the “cooperative autocatalysis” approach [18]. Furthermore, it is able to explain the kinetics of spontaneous generation [18], the association between infectivity and aggregated PrP, the linear appearance of the fibrils and to take into account fundamental processes of an *in vivo* replication (i.e. fibrils splitting), all while remaining relatively mathematically tractable. Moreover, its dynamical behavior has been extensively studied [21],[22], and experimental measurements were used in [14] to provide an estimation of the full set of parameters for a particular prion strain. The model has three state variables (Eq. 10) describing the amount of monomer (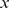), polymer (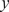) and the mass of polymer (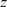), and it involves 6 parameters (see Table 1). We reproduce here only the features essential to discuss the strain dependence of its parameters; the details are covered in Materials and Methods.

**Table 1:** Model symbols.

**Model state variables**	amount of monomer	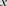
	amount of polymer	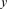
	mass of polymer	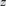
**Model parameters**	nucleus size	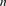
	rate of monomer production	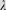
	rate of degradation	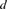
	rate of aggregation	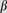
	rate of clearance	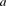
	rate of breakage	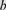

Description of all state variables and parameters.

In [8] it has been shown that for any prion strain two parameters, the rate of growth (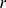) and the reproductive ratio (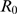), can be estimated from experimental data. The former (Eq. 2) represents the exponential growth of the number of infectious particles. The latter (Eq. 3) is defined as the average number of prion fibrils that a single infectious particle can give rise to, before splitting into fibrils smaller than a critical size or being degraded. In other words, 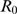 represents the ability of the fibrils to survive to critical breakage and degradation events. The equations for the 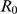 and 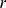 parameters obtained from the kinetic model of [8] can be reparametrized in terms of the mean length of the fibrils 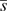


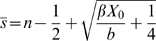
(1)obtaining:
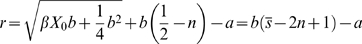
(2)

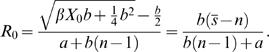
(3)


**Figure 1:**
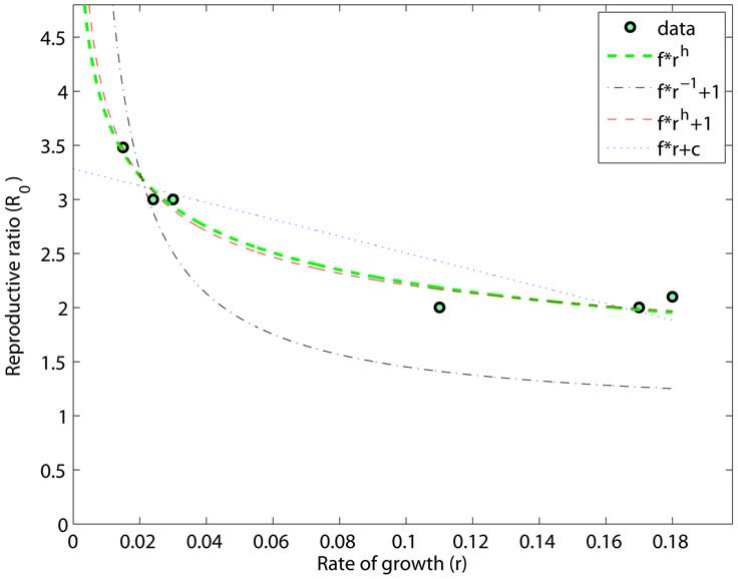
Relationships between the empirical parameters 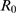 and 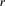. The reproductive ratio is plotted against the rate of growth. The downward trend is not well described by the linear model with negative angular coefficient (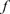) and an intercept (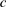) (dotted blue line). In addition, the model prediction with 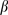 fixed (dashed-dot black line) fails to precisely represent the data, even if it provides a more reasonable relationship (notice that high stable prions, such as MK4985, would always be associated to positive 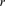 values). Introducing one more degree of freedom (exponent 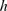) yields a higher 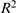 value (red line, 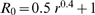). This result corresponds to a prediction of 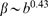. In addition, we tested a further simplified model version (where 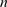 is considered to be much smaller than 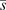) according to which 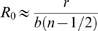 (i.e. 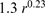, shown in green). Similar conclusions could be drawn.

In order to estimate from experimental measures both parameters (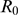 and 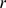) certain assumptions are necessary (see Materials and Methods for full details). An estimation of 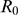 and 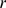 from *in vivo* experiments and for different prion strains characterized by different values of stability against denaturation (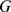) is listed in Table 2. The dataset currently available is limited (as not many prion strains can be fully characterized) and many error sources are potentially affecting the estimation of the parameters. Nevertheless, Figure 1 shows the existence of a negative trend between these two empirical parameters (Pearson correlation = −0.91, p-value = 0.01). If we now turn to the kinetic model and look at the corresponding expressions (Eq. 2, 3) the interesting question is whether such a behavior is predicted by the model itself, and is explainable in terms of some of its parameters, in a way that is both mathematically and biologically plausible. Otherwise stated, we investigate which, if any, among the model parameters best describe the strain variability. The critical size of the nucleus (parameter 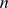 in the model) plays a marginal role in our analysis and is likely to be a fixed integer, in between 2 and 4, across different strains [23]. Even though it has been argued that a hexamer is the minimum infectious unit [24], it can be shown that the model-based conclusions are not conditioned by the value of 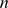. In addition 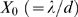 is clearly independent of the prion strains, so we remain with three possible choices: 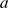, 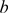 and 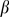. From Eq. 1, increasing 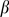 means incrementing 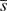 and this affects 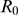 and 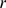 in a similar manner, so that this parameter alone cannot explain the inverse relationship derived in Figure 1. The same can be said for 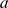 and 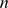 which, if increased/decreased, would induce changes of equal sign in 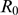 and 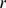. Different conclusions can be drawn when considering 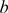 as the only strain-varying parameter. This dependence becomes clearer assuming that fibrils cannot be degraded in the exponential phase (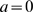, identical results can be obtained supposing that the degradation of the fibrils scales as the fibrils breakage rate, 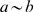, see Text S2). Such assumption leads to the following expressions:
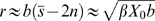
(4)

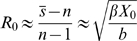
(5)

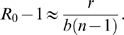
(6)


**Table 2:** Estimated empirical parameters for different prion strains.

Prion strain	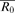	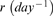	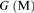
139A	–	0.05 [34]	2
ME7	3	0.024	2.9
BSE	3.48 [35]	0.015	2.8
Sc237	2 [36]	0.11 [37]	1.6
RML	2.1 [14]	0.18 [38]	1.7
MK4985	3.9	–	3.8
vCJD	–	0.07 [39]	1.85 [40]
Fukuoka-1 CJD	3 [41]	0.03 [42]	–
Chandler Scrapie	2 [8]	0.17 [8]	2.2
301 V	–	0.07 [43]	2.2

The estimated values for the reproductive ratio (Eq. 11), rate of growth and stability against denaturation for different prion strains are shown. One of them (MK4985) is a synthetic prion strain that requires a high concentration of Gdn-HCL to denature 50% of the pathogenic protein. Whenever no reference is shown, [3] is used.

If we keep into account only the dependence from 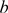, then Eq. 4 and Eq. 5 can be simplified to
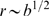
(7)

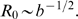
(8)


From these simplified formulas it is clear that an increase in the frangibility of the fibers (i.e., in 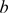) produces an increment of 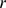 (Eq. 7) and a decrement of 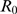 (Eq. 8) in agreement with the trend in Figure 1. Therefore, from the model we expect 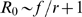 to give the best fitting result. As a matter of fact, this relationship (black dash-dotted line in Figure 1) does not provide the optimal fit, although it reproduces the qualitative observed behavior (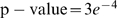). The fittings of Figure 1 (see Table 3) suggest that, approximately, 
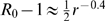
 (red line) implying that we are observing 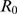 proportional to 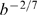 and 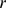 to 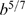 (see Materials and Methods, Eq. 12). This means that the estimated exponents for 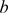 are somewhat different from the expected values of (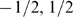) predicted in Eq. 7 and 8. In order to improve the model prediction, we introduce a strain-dependence on a second parameter. The simplest solution suggested by the model for this scope (deducible from Eq. 4 and 5) points to the aggregation rate 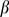. By linking 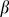 to 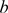, we are still left with a one-parameter family of models describing the strain-dependence. In doing so, we obtain the estimate 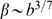 (see again Materials and Methods, Eq. 13). This correction yields 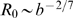 and 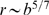, this time respecting the predictions of Eq. 4 and 5. Therefore, on the one hand we can show that at a qualitative level 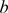 is the only parameter that alone can explain the inverse relationship between 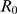 and 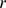. On the other hand, the variation of 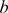 by itself is not able to quantitatively describe the experimental data in a precise way. An additional correction, obtained relating 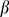 to 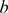, leads to a substantially improved fitting. Apart from Eq. 4 and 5, our choice of 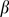 alongside 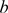 as strain-dependent parameter is suggested by the structure of the model of Eq. 10, in which, of all parameters, those describing the kinetics of fibril aggregation/breakage are the most likely to vary across strains. Both the fitting and the model structure suggest an interplay between 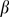 and 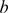, with 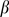 partially balancing the effect of 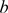.

**Table 3:** Fitted values for the curves in Figure 1 and Figure 2.

Relationship	Estimated parameters	R-square	p.value
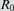 **vs** 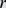	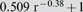	**0.97**	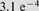
	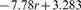	0.83	0.0109
	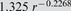	0.96	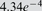
	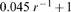	0.04	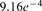
	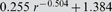	*0.97*	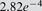
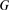 **vs** 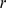	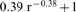	**0.69**	**0.01**
	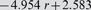	0.41	0.0848
	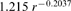	0.67	0.0123
	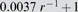	0.53	0.0104
	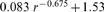	*0.7*	*0.0093*
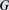 **vs** 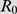	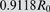	0.87	0.0069

The linear and non linear relationships, with and without the intercept, for 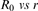 and 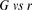 are reported here. These models are fitted to the experimental measurements listed in Table 2. For each model the fitting parameters, 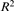 and the correlation p-value are reported. When 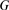 and 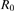 are related to 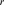, the non linear model with a fixed intercept and a free exponent (i.e. 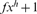) is associated with the best fitting results (bold). By adding one more free parameter (i.e. 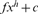) we do not get essentially any improvement (italic). The estimated 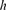 value for 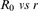, without any simplification, implies 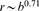, 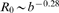 and 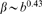 (see Materials and Methods). A direct proportionality is observed also for 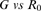.

**Figure 2:**
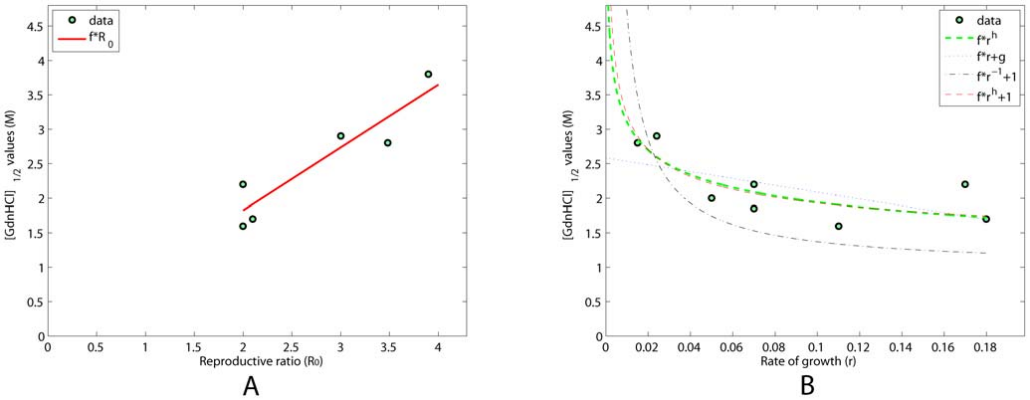
Relationships between 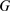, 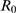 and 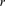. In (A) and (B) the stability against denaturation is plotted against the reproductive ratio and the rate of growth. A direct proportionality links 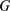 to 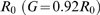. As expected, an inverse proportionality emerges between 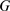 and 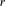, reinforcing the previous results.

In the following, we will describe how the previous results can be extended to the stability to denaturation of the prion strains, providing experimental observations in support of our claims. From Figure 2A a direct linear proportionality between 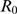 and 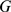 is inferred. Therefore, combining the fitting between 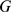 and 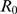 and 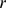 and 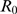, a similar inverse relationship (see Figure 2B) relates 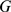 and 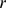 (see Table 3). A point of note is that a linear model (i.e., 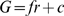) is not only associated to a low coefficient of determination 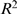 but is also implausible, as it predicts negative values of 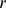 in correspondence of very stable prion strains (such as MK4985, see Table 4). Owing to the linear proportionality (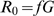) of Figure 2A, the inferred functional dependencies from 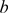 extend to 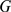 (i.e., 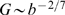). This result, in light of the experimental observations in [10], contributes to validate the results of the kinetic model and provides us with a simple practical tool to interpret prion strain stability. As a matter of fact, the experimental data in [10] report a relationship between the chemical stability of yeast prion strains and their structural properties, hence reinforcing our conclusions. In particular, the frangibility of different Sup35NM amyloid conformations was measured and shown to be consistent with an increase in sensitivity to denaturants and proteases. Thus, confirming the main role of the breakage rate, as predicted here by the model. Furthermore, the authors observed also a variation in the aggregation rate (parameter 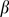 in the model), which was however overcome by the stronger effect of the division rate; an additional observation in agreement with our results, where the best match with the experimental data is obtained for a variation of 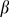 that only partially compensates for that of 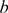. The importance of breakage events for the *in vivo* prion propagation is also underlined in [25], where the authors observed that membrane-anchored PrP is necessary for the exponential growth of prion aggregates. In transgenic mice, expressing anchorless prion protein inoculated with different prion strains, the aggregates seem to grow quadratically in time [26]. This feature is explainable by a linear aggregation model (i.e setting 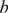 equal to 0). Moreover, in [26], different prion strains show a common inability to induce the disease. The absence of fibrils disruption can prevent the formation of oligomeric species, thus hiding the difference between prion strains. Our model-based analysis suggests that an experiment monitoring the propagation of prion strains lacking the GPI anchor would be useful to characterize in more depth the strain phenomenon.

**Table 4:** Estimated model parameter 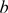 for different prion strains.

Prion strain	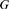	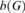	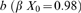	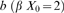	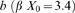	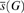
139A	2	0.0352	0.033	0.0665	0.1131	7.39
ME7	2.9	0.0036	0.015	0.0316	0.0538	9.36
BSE	2.8	0.0043	0.017	0.0339	0.0577	9.14
Sc237	1.6	0.2132	0.051	0.1039	0.1767	6.51
**RML**	**1.7**	**0.1241**	**0.02**	**0.06**	**0.15**	**6.73**
MK4985	3.8	0.0009	0.009	0.018	0.031	11.33
vCJD	1.85	0.0625	0.038	0.078	0.132	7.06
Chandler Scrapie	2.2	0.0184	0.027	0.055	0.093	7.83
301 V	2.2	0.0184	0.027	0.055	0.093	7.83
RecMoPrP (89–230)	5.1 [3]	0.0002	0.005	0.010	0.017	14.19

Using Eq. 9 and assuming 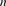 equal to 3, the breakage rate can be estimated (second column, 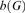) from G. In [14] the authors provide for the RML strain (bold) a lower and an upper bound for 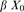 (0.98 and 3.4 prion/day) in addition to the best estimate (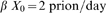). The fitting obtained in Figure 2A is used here to infer 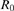 from 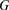. We can fix 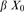 to the values reported in [14] for different strains and estimate 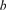 (as the only varying parameter) from Eq. 11. Comparing the 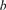 values estimated in [14] and our extrapolated values, we see contained differences (see Figure S1). This result shows that 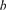 is the main parameter explaining strain kinetic variability. A remarkable advantage of this method is that it requires only a single rather simple experimental measurement (i.e. resistance to guanidine denaturation) in order to predict the replication dynamics of a particular strain.

In the last part of the Section, we investigate how the prion stability (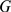) is reflected in the mean length of the fibrils (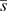). Combining the fitting of Figure 2 with Eq. 6, 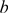 (and consequently 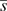, from Eq. 1) can be inferred directly from 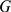 and 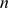:
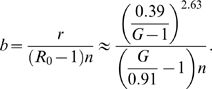
(9)In Table 4, we compare the approach of Eq. 9 with the results obtained in [14], where the authors give a complete estimation (including a range of uncertainty) of all the parameters for the RML prion strain (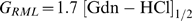, highlighted in bold in Table 4). The comparison between these two approaches shows that the predictions obtained through Eq. 9 are similar to the values reported in [14] for the RML strain. In addition, we can compare the values of 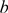 for the strains inferred from Eq. 9, with the ones computed using Eq. 11 and then imposing 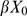 equal to the values of [14] for the RML strain (see Figure S1). Our predictions are approximately within the range of values computed considering 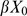 constant among strains. This result reinforces the major role of 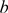 in explaining strain variability. Owing to the fact that 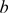 is now strain-dependent (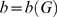), we can also predict the mean length of the fibrils (Eq. 1) for each considered strain (see Table 4, 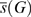). For instance the mean length of the fibrils population for two prion strains with different stabilities (e.g. RecMoPrP (89–230) and Sc237) can be compared. For the unstable prion strain (Sc237) this is approximately 7 monomer units, while for the stable prion strain (RecMoPrP (89–230)) it is approximately 14 monomer units. This theoretical approach provides a valuable method to simplify the model characterization. Furthermore, it contributes to understanding the properties associated to prion strains with different stability against guanidine denaturation.

## Discussion

While it is reasonable that the parameters of the kinetic model might all be affected by strain specificities (i.e. stability against denaturation), the dominant contribution seems to be due to the susceptibility to frangibility (i.e. 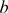), with only a minor correction due to 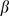. The inverse relationship between 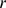 and 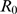 shown in Figure 1 is the main argument in the identification of 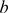 as the key physical aspect differentiating prion strains. In addition, 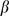 is suggested as the most plausible and parsimonious correcting factor, in order to improve the data fitting.

Several aspects can influence the estimation of the parameters and the model predictions. For example, the uncertainty affecting the estimation of 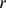 and 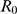 (respectively inferred from an exponential curve and from a ratio of exponentials); or the possibility that the breakage rate is not equal across all the different polymer lengths (e.g. mechanical stress can differently affects longer fibers); or even the impact of the mouse age on the model parameters (affecting e.g. the 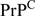 production rate). In spite of these (and potentially many other) disregarded aspects characterizing an *in vivo* system, this simple model is able to capture and explain the observed data dependencies through arguments supported by multiple independent experimental observations. Our analysis reveals that stable prion strains can be characterized by a “stronger” aggregated structure which is less prone to breakage events. This will further imply a longer mean length of the fibrils. Instead, unstable prion strains are subject to a higher fragmentation rate. The role of 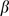 is essentially to partially balance the increased breakage and is coherent with the experimental observations in yeast. Furthermore, the increased number of catalytic sites may be also responsible for the shorter incubation time.

As already mentioned, such phenomenon was observed in yeast prions [27]. The yeast prion proteins, although fundamentally different from the mammalian prion proteins, show the same ability to convert into aggregated forms, propagate and be infectious. This simpler unicellular system is a valuable model as it enables a deeper analysis of the fibril formation process [28], not possible to the same extent in higher organisms.

The framework proposed allows for a model-based analysis of these properties in mammalian prions *in vivo*. In the context of mammals, our results are consistent with [9], where fibrils with different conformational stability are generated *in vitro* from full length mammalian PrP. In that paper, the authors relate the stability to the size of the smallest possible fibrillar fragment without taking into account the kinetics of the replication (reproducing the *in vivo* behavior). We draw similar conclusions from a different point of view. As a matter of fact, we investigate the dynamic evolution of prion propagation in a multicellular *in vivo* system, in which molecular and cellular mechanisms are present as well. Our model-based conclusions provide further evidence that *in vitro* systems and yeast prion propagation mechanisms can be transposed in mammals. Moreover, linking the strain phenomena to dynamical features leads to a characterization of the evolution of the length of the fibrils *in vivo*.

**Figure 3:**
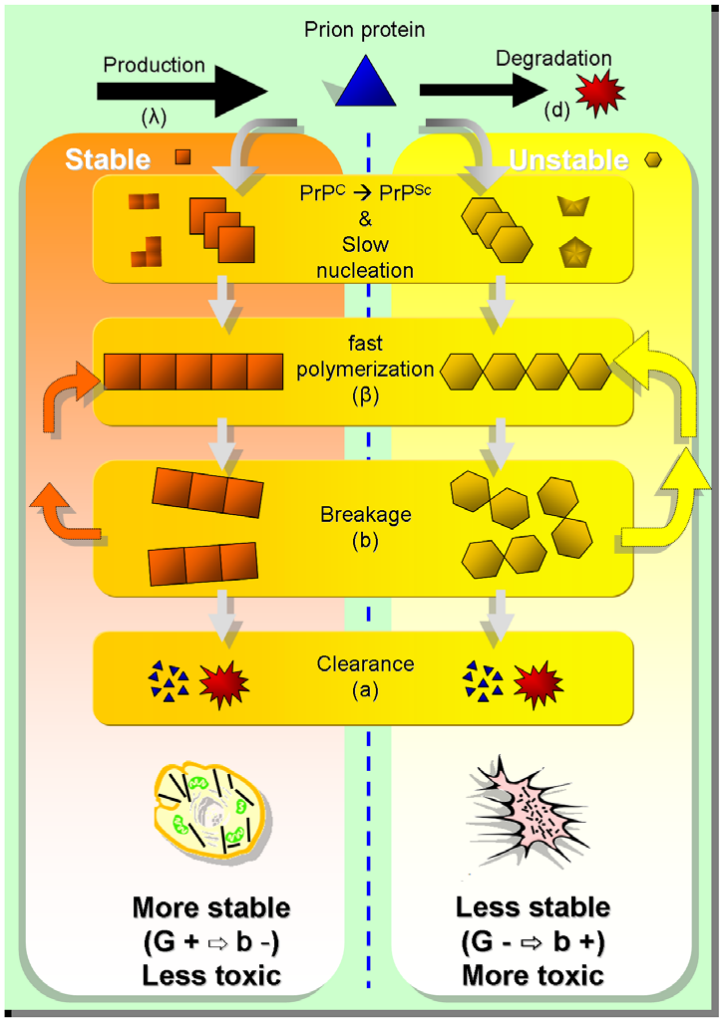
Kinetic model and prion pathways. The cartoon describes the pathways of kinetic replication of two prion strains with a different stability against denaturation: a stable one (high 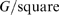) and an unstable one (low 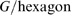) are drawn. These act as templates bringing the same cellular prion protein (triangle) to the two different strain conformations (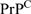 ▴→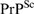 ▪, ♦). The model assumes that the aggregation of monomers to polymers produces a very fast change of conformation and that this aggregation is unfavorable below a critical size (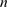), which is assumed to be independent of the prion strain in our model. The experimental data suggest that stable prions are characterized by a higher 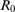 and a corresponding lower 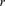. In the model, this is translated into strain-specificity of the rates of breakage and of aggregation (which are both lower for stable prions). This implies that stable fibrils are longer and prefer to proliferate while maintaining themselves as fibrils larger than the nucleus size (pathway on the left). On the contrary, unstable prions are more frangible (i.e. more sensitive to breakage), implying a shorter mean length. This means that breakage events are more likely to be associated with the formation of very short fibrils, even under the critical size. The increase in the aggregation rate is not enough to avoid an increased growth in the number of fibrils. We can therefore hypothesize that an apoptotic pathway is most likely for these last strains (pathway on the right). These conclusions are in agreement with the working hypothesis of oligomer toxicity [44].

We can, in fact, speculate (in agreement with [5]) that stable prion strains exhibit a proliferation of longer fibrils that, upon splitting, still manifest the same stability properties (Figure 3), giving rise to a preferential proliferation of relatively long fibrils with a low toxic effect. On the other hand, less stable prion strains tend to form shorter fibrils, to proliferate faster and to be more neurotoxic.

It is worth noting the connection with [13], where the kinetics of aggregation of amyloid peptides is studied by means of coarse-grained molecular dynamics. The authors showed how the relative stability of 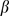-prone states of a polypeptide can influence the pathway of aggregation. Their results suggest that the 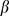-stable amyloids follow an aggregation pathway without intermediates, while 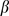-unstable amyloids seem to involve on-pathway oligomers.

The characterization of prion strains in terms of polymer mean size is *per se* a significant observation. It provides a new possible explanation of the observation that stability is correlated with lesion profiles and vacuolation areas. Several hypotheses have been made, such as the existence of a co-factor that supports the conversion of distinct prion strains in precise brain regions. Here, another possibility emerges: the increased size associated to stable prions can decrease their ability to diffuse, and can circumscribe them to small brain regions. On the contrary, oligomers can spread around the brain more easily, causing a more homogeneous damage.

In conclusion, we show that linking the conformational stability property of prions, acquired during *in vivo* propagation in mammals, to their replication kinetic properties is achievable through a rather simple model. For a wide range of parameters, the model predicts that a higher breakage rate 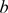 implies shorter 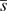 and shorter incubation time (in Figure S2 two simulations are compared). Our model-based approach suggests that the amount of information that can be extrapolated from the knowledge of 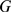 goes beyond the expected incubation time.

## Materials and Methods

### Kinetic model

*In vitro* prion propagation is characterized phenomenologically by the following properties: (i) a critical concentration threshold below which fibrils cannot form; (ii) a delay before their propagation (which can be eliminated by the addition of seeds of preformed fibrils); (iii) a direct proportionality between the initial rate of fiber growth and the monomer concentration [29]. The overall behavior resembles a sigmoidal growth curve [30]: an exponential growth of infectious particles followed by a plateau. The simplest description of the underlying observed mechanism of protein aggregation consists of a slow continuous nucleation followed by a fast autocatalytic growth. Therefore a simple two-step model is able to reproduce the dynamics of the *in vitro* prion propagation [12]. An *in vivo* prion propagation model should explain the fact that the spontaneous prion-induced disease is rare but progresses inevitably after infection, that the incubation period is long and followed by a brief fatal clinical disease and that prions undergo several molecular processes within the cell (e.g. fibrils breakage, degradation, endogenous 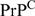 production). The model derived in [8] is obtained as a closed form of an infinite set of differential equations describing the variation in time of the monomer and fibrils of each possible length (from 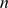 to 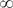). The biological mechanisms taken into account are the lengthening at the fiber end by the addition of monomers, the degradation of polymers, and their splitting into smaller polymers. Only if several monomeric 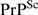 molecules are mounted into a highly ordered seed, can further monomeric 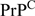 be recruited and form amyloid aggregates. If, after the breakage, the fibril has a length under the critical size, it degrades instantaneously into normal 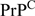 monomers. The model in Eq. 10 has three state variables, describing the amount of monomer (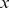), polymer (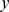) and the mass of polymer (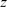), and it comprises of 6 independent parameters: nucleus size (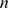), rates of production (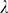), degradation (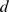), aggregation (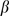), clearance (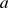) and breakage (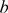):
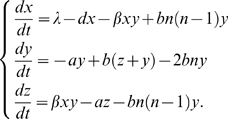
(10)


The assumption that 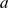 is negligible, made in 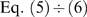 in order to simplify the parameters equations, changes the qualitative behavior of the model, that no longer has two stable steady states but only one, which is unstable. This means that the exponential growth will never reach a plateau. As mentioned in the text, this does not affect our previous considerations, especially in light of the fact that *in vivo* death occurs during the exponential growth phase (see also the Text S2 for similar conclusions on the full model).

### Measuring the parameters 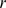 and 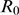


In this section we summarize the procedures mentioned in [8] and adopted here to derive a measure for 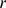 and 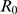. The assumptions deemed, in order to measure 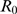 and 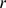 from the observed effect of different levels of PrP expression and inoculum dosage, are as follows:

i) The linear relationship relating the incubation time to the inoculum log dilution reflects the exponential growth of the infectious units.
ii) The only parameter that varies between two transgenic mice with an altered level of 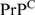 expression is considered to be the 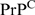 production rate (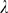).
iii) The termination stage (animal death) occurs during the exponential growth phase.
iv) The level of 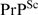 in the brain at the termination stage can be considered to be the same in all experiments.

Of all assumptions, the last one is the most important. It is considered valid also for transgenic mice expressing different quantities of cellular prion protein. Currently there is wide debate about the cause of cell death in prion neurodegeneration. From knockout mutants, it seems that 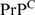 loss of function is not sufficient to cause cell death. What has been observed is that the conversion of 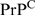 to the 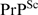 isoform has a key role in the disease. In spite of their apparent low neurotoxic effect [26], fibrils have been proven to be the main ingredient in catalyzing variations of protein conformation [31]. Therefore, it is reasonable to assume that even if toxicity is not directly associated to fibrils aggregates, it has to be closely related to their amount, implying that a critical concentration of 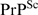 is required to provoke cell death. The current working hypothesis is that oligomeric species are the most infectious [32] and a substantial body of evidence suggests that they are also highly cytotoxic [33]. According to the previous observations, a possible explanation is that an equal mass of prion fibrils with smaller mean size provides a larger number of active sites for catalysis, hence inducing a higher lethality.

In order to extrapolate a measure for 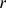 we follow the method described in [8] based on relating the incubation time 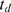 to the inoculum dose and implying an exponential growth in the number of infectious particles. Taking advantage of these data (e.g. incubation time 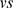 inoculum dosage), we can infer the 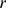 parameter just by fitting an exponential growth curve. More precisely, before inoculation of prions, PrP (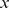) can be considered at steady state (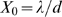). After inoculation, it is reasonable to assume that it remains almost constant for a while. According to the model equations, the steady state of the mean polymers distribution length (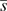 in Eq. 1), is typically reached before the exponential phase. Immediately after reaching 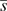, the polymer amount (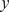) and the polymer mass (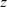) start to grow exponentially. Thus, 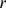 is defined as the dominant mode of this exponential growth (i.e., 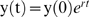) (Eq. 2).

To have an indirect measurement of 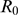, the inverse relationship between incubation time 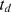 and the PrP expression is exploited. We take into account the previous assumptions reporting that the number of infectious units in two inoculated mice expressing different level of PrP (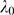, 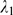) at the times of death (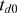, 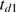) can be considered almost equal. Thus imposing 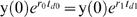 we can derive 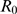:
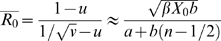
(11)where 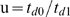 and 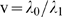.

For a more detailed description see Appendix of [8]. It is worth noticing that the incubation times listed in [3] are not the same as those used to estimate 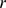 (see Text S1 for more details).

### Computing the 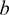 and 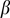 exponents

Rather that using Eq. 7 and 8, the exponents 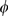 and 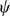 such that 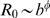, 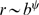 can be computed from the fitted curve in Figure 1. We approximate the numerical value 0.38 of Table 3 with 0.4 (i.e. 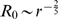). From the above expressions, 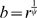, which yields 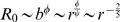, i.e., 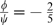 or 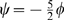. Examples of values on this line are:
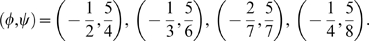
From Eq. 6 it is clear that the only admissible pair of values is
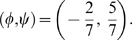
(12)If, following Eq. 4 and 5, we add the extra functional dependence of 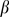 from 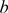 as 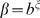, we can look for a value of 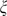 that satisfies simultaneously
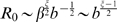


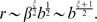
yielding:
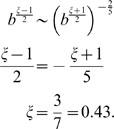
(13)


## Supporting Information

Figure S1 Plot of Table 4
(0.03 MB PDF)

Figure S2 Disease evolution for different values of b
(0.05 MB PDF)

Text S1 Incubation time (t_G) vs stability (G) and rate of growth (r)
(0.12 MB PDF)

Text S2 Full model considerations
(0.04 MB PDF)
