# Supplementary material for: Investigating the Conformational Stability of Prion Strains through a Kinetic Replication Model
Source: PLoS Comput Biol. 2009 Jul 3;5(7):e1000420. doi: 10.1371/journal.pcbi.1000420 (PMC2697384; doi:10.1371/journal.pcbi.1000420)
Supplement: Figure S1 — Plot of Table 4 (0.03 MB PDF) [file pcbi.1000420.s001.pdf]

Plot of Table 4

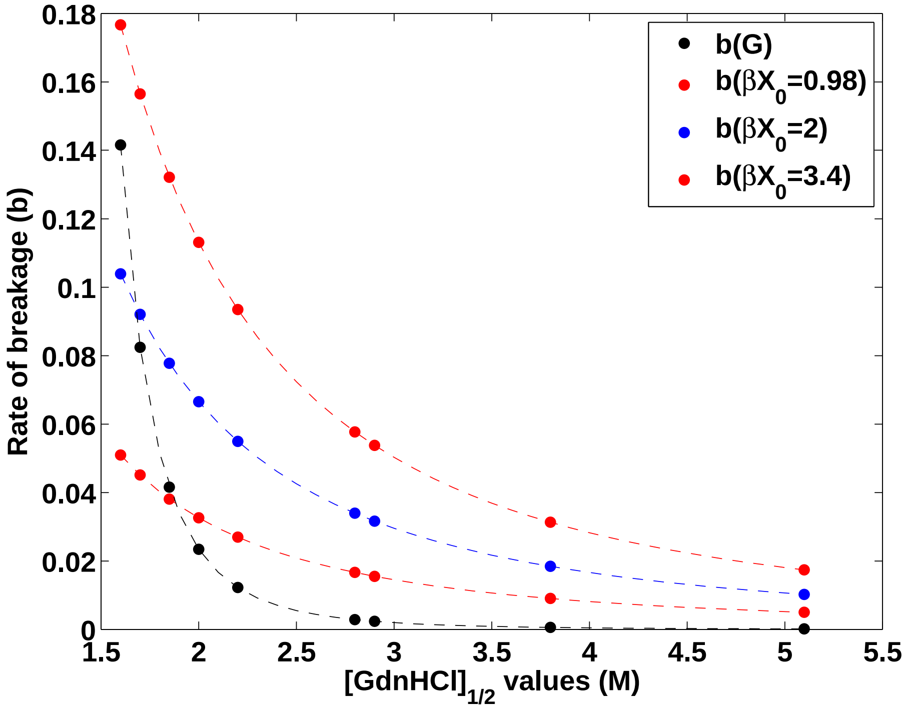

Figure S1: **b(G) and b(βX_0):** Results obtained in Table 4 in the main text are here visualized.
