# Supplementary material for: Investigating the Conformational Stability of Prion Strains through a Kinetic Replication Model
Source: PLoS Comput Biol. 2009 Jul 3;5(7):e1000420. doi: 10.1371/journal.pcbi.1000420 (PMC2697384; doi:10.1371/journal.pcbi.1000420)
Supplement: Figure S2 — Disease evolution for different values of b (0.05 MB PDF) [file pcbi.1000420.s002.pdf]

Disease evolution for different values of b.

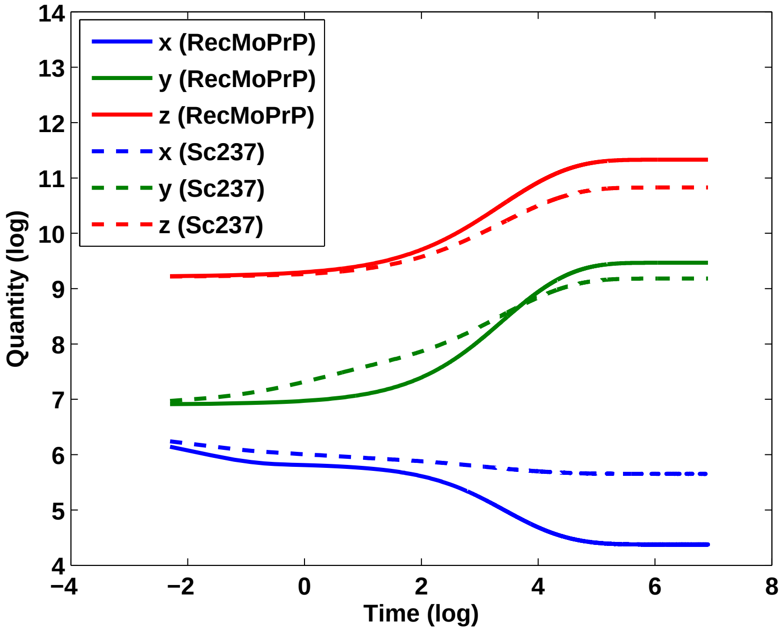

Figure S2: **Simulation of disease progression for different values of b.** We selected the same strains reported in the "Result" Section of the paper. The corresponding solutions of the ODEs in Eq. 8 of the main paper are shown (solid for RecMoPrP, dashed for Sc237). As expected, the associated disease steady states have different fibril compositions, and the incubation times are also different. From this figure the effect of a variation of b is evident also on the replication dynamics.
