# Supplementary material for: Investigating the Conformational Stability of Prion Strains through a Kinetic Replication Model
Source: PLoS Comput Biol. 2009 Jul 3;5(7):e1000420. doi: 10.1371/journal.pcbi.1000420 (PMC2697384; doi:10.1371/journal.pcbi.1000420)
Supplement: Text S1 — Incubation time (t_G) vs stability (G) and rate of growth (r) (0.12 MB PDF) [file pcbi.1000420.s003.pdf]

Incubation time (t_G) *vs* stability (G) and rate of growth (r)

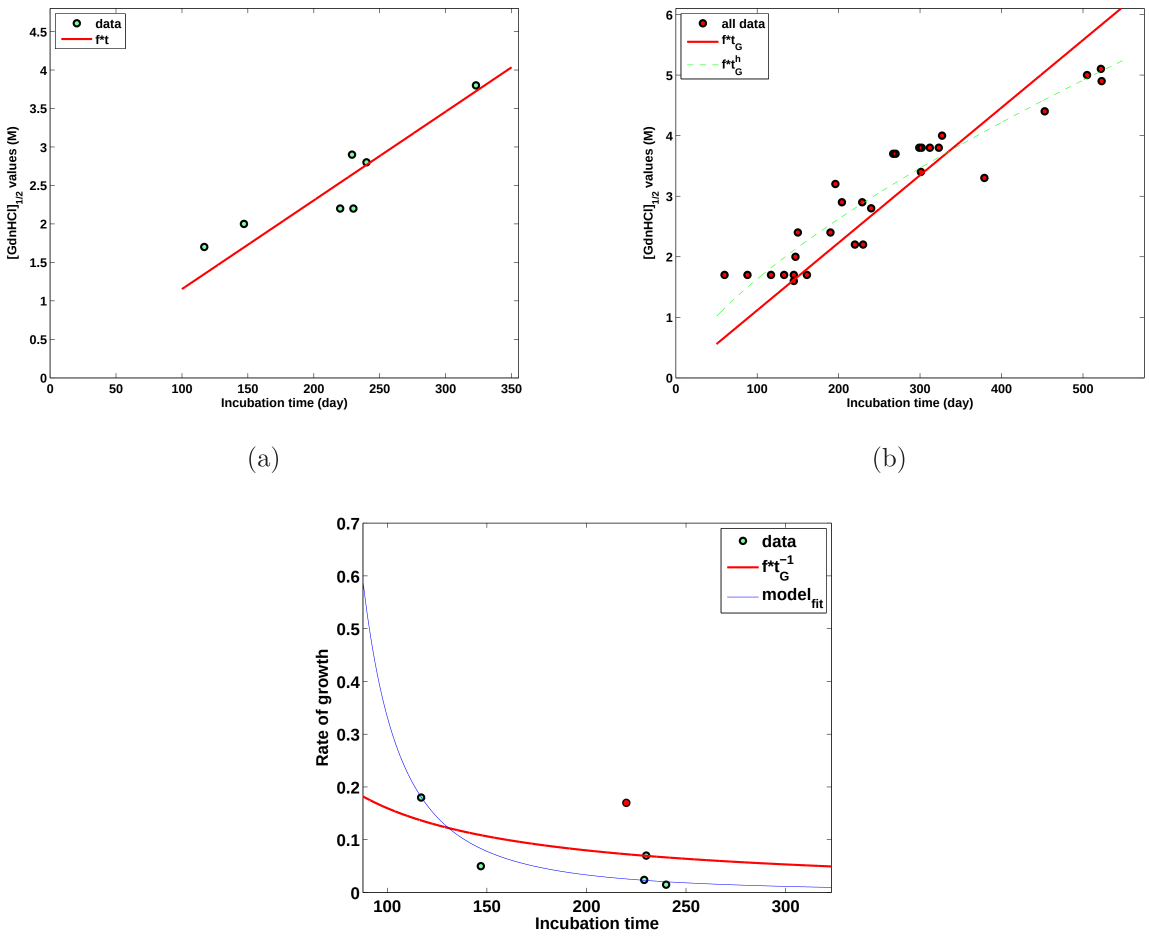

(a) (b)

(c)

Incubation time (t_G) *vs* stability (G) and rate of growth (r).

In [1] a direct linear proportionality between the resistance of different strains to denaturation (G) and the incubation time (t_G) is reported. In Fig. S0 (a) we repropose this linear dependence between G and t_G for the strains listed in Table 2 of the main manuscript (see Table S1). The more stable a prion is, the longer is its incubation time. Synthetic prions, provide us with the opportunity to explore this relationship in a wider range of stability values than when considering only natural prion strains [2]. The regression of the entire dataset reported in [1] (see panel (b)) reveals that the linear (red line) and the non linear (dashed green line) model have comparable fitting performances, see Table S2. Natural and synthetic strains separately are directly proportional to their respective incubation times t_G (see again [1]). However, the best fitting of the combined dataset is obtained for the non-linear model

(green dashed line, Table S2). As the highly stable strains have long incubation times, also the age factor should probably be taken into account. The t_G of Fig. S0 (a) are clearly function of G and hence provide information on the growth rate of each strain. However, the t_G of [1] do not carry any information about inoculum dose, and as such cannot be directly used to infer r according to the method described in [3] (see also the Material and Methods section of our manuscript). For this reason, we decided to use the values of r (and R_0) available from the literature, not based on the t_G of [1]. In panel (c), then, we investigate the relationship between these stability-based incubation time t_G, and the estimated rates of growth r (Table 2 of the main manuscript). Qualitatively, an inverse relationship is emerging, as expected. However, this relationship is not exactly of the type occurring between r and t_d ($r \sim 1/t_d$). In Table S2 we show that this particular relationship is not "optimally" reproducing the experimental data, as can be anticipated by concatenating the various best fitting carried out in the paper: R_0 from r, G from R_0 (and finally here G from t_G). Relating r to t_G through this chain of models, we obtain an improved fitting, although still below the level of significance (p-value is in Table S2). This may be due to the presence of an outlier (marked in red). Neglecting this outlier, the model predictions are associated to a p-value less than 0.05 (p-value=0.0318), while the simpler inverse relationship ($r \sim 1/t_G$) is still below the level of significance (p-value=0.0742).

Prion strain	G (M)	Incubation time t_G (days)
139A	2	147
ME7	2.9	229
BSE	2.8	240
RML	1.7	117
MK4985	3.8	323
Chandler Scrapie	2.2	220
301V	2.2	230

Table S1: **Estimated empirical parameters for different prion strains.** The estimated values for the stability against denaturation and the incubation time t_G for different prion strains are shown. Notice that the incubation times t_G are not corresponding to those used to infer r and R_0 (denoted t_d in the manuscript).

Relationship	Estimated parameters	R-square	p.value
G **vs** t_G **(a)**	$0.0114\ t_G$	0.84	0.004
G **vs** t_G **(b)**	$0.0696\ t_G^{0.685}$	0.874	$4.12e^{-14}$
	$0.01115\ t_G$	0.724	$8.32e^{-14}$
r **vs** t_G	$(0.107\ t_G^{0.685} - 1.4112)^{-2.63}$	-	0.2353
	$15.99/t_G$	-	0.3374

Table S2: **Fitted values for the curves in Fig. S0**

References

[1] G Legname, H O Nguyen, D Peretz, F E Cohen, S J DeArmond, and S B Prusiner. Continuum of prion protein structures enciphers a multitude of prion isolate-specified phenotypes. *Proc Natl Acad Sci U S A*, 103(50):19105–19110, Dec 2006.

[2] G Legname, I V Baskakov, H O Nguyen, D Riesner, F E Cohen, S J DeArmond, and S B Prusiner. Synthetic mammalian prions. *Science*, 305(5684):673–676, Jul 2004.

[3] J Masel, V A Jansen, and M A Nowak. Quantifying the kinetic parameters of prion replication. *Biophys Chem*, 77(2-3):139–152, Mar 1999.
