# Supplementary material for: Investigating the Conformational Stability of Prion Strains through a Kinetic Replication Model
Source: PLoS Comput Biol. 2009 Jul 3;5(7):e1000420. doi: 10.1371/journal.pcbi.1000420 (PMC2697384; doi:10.1371/journal.pcbi.1000420)
Supplement: Text S2 — Full model considerations (0.04 MB PDF) [file pcbi.1000420.s004.pdf]

Full model considerations

It is worth to notice that even avoiding the simplifications in eq. 4-5 of the paper, at least qualitatively we can still draw the same conclusions as in the paper. In fact, for a particular range of the parameter b we still get an inverse relationship between R_0 and r, even if fitting all experimental data just by varying b becomes less straightforward. The claim is that prion strains can be fully characterized just by an inverse "stability vs. breakage ratio", as derived from the simplified model structure. Considering the full expression of r and R_0, according to Eq. 2 and 3 of the paper it is possible to find an interval of b values for which the derivative of R_0 with respect to b is negative, the derivative of r positive, and the system solution converges to the steady state representing infection. The inequalities in Eq. 1 represent, respectively, the conditions on the derivative of R_0, of r and on the parameters set.

$$\begin{cases} \dfrac{dR_0}{db} = \dfrac{-\frac{1}{2}b^2\sqrt{\frac{\beta X_0}{b}}(n-\frac{1}{2}) + \frac{1}{2}a\sqrt{\frac{\beta X_0}{b}}}{(a+(n-\frac{1}{2})b)^2} < 0 \\ \dfrac{dr}{db} = \dfrac{1}{2}\left(1-2n+\dfrac{b+2\beta X_0}{\sqrt{b(b+4\beta X_0)}}\right) > 0 \\ 0 < X_0 - \dfrac{(a+(n-1)b)(a+nb)}{\beta b}. \end{cases} \tag{1}$$

The inequalities of Eq. 1 are simultaneously satisfied in the following intervals:

$$\begin{cases} b > b_1 \\ b < b_2 \ \ or\ \ b > b_3 \\ b_4 < b < b_5 \end{cases} \tag{2}$$

where

$$b_1 = \frac{a}{n-\frac{1}{2}} \tag{3}$$

$$b_{2,3} = \frac{-2\beta n(n-1)X_0 \pm \sqrt{\beta^2(1-2n)^2(n-1)nX_0^2}}{(n-1)n} \tag{4}$$

$$b_{4,5} = \frac{a-2an+\beta X_0 \pm \sqrt{a^2+2a\beta(1-2n)X_0+\beta^2X_0^2}}{2n(n-1)}. \tag{5}$$
